# Molecular Profiles of Multiple Antimalarial Drug Resistance Markers in *Plasmodium falciparum* and *Plasmodium vivax* in the Mandalay Region, Myanmar

**DOI:** 10.3390/microorganisms10102021

**Published:** 2022-10-13

**Authors:** Hương Giang Lê, Haung Naw, Jung-Mi Kang, Tuấn Cường Võ, Moe Kyaw Myint, Zaw Than Htun, Jinyoung Lee, Won Gi Yoo, Tong-Soo Kim, Ho-Joon Shin, Byoung-Kuk Na

**Affiliations:** 1 Department of Parasitology and Tropical Medicine, Institute of Health Sciences, Gyeongsang National University College of Medicine, Jinju 52727, Korea; 2 Department of Convergence Medical Science, Gyeongsang National University, Jinju 52727, Korea; 3 Department of Medical Research Pyin Oo Lwin Branch, Pyin Oo Lwin 05062, Myanmar; 4 Department of Tropical Medicine, Inha Research Institute for Medical Sciences, Inha University College of Medicine, Incheon 22212, Korea; 5 Department of Microbiology, Ajou University College of Medicine, Suwon 16499, Korea

**Keywords:** *Plasmodium falciparum*, *Plasmodium vivax*, drug resistance genes, malaria, Myanmar

## Abstract

Emergence and spreading of antimalarial drug resistant malaria parasites are great hurdles to combating malaria. Although approaches to investigate antimalarial drug resistance status in Myanmar malaria parasites have been made, more expanded studies are necessary to understand the nationwide aspect of antimalarial drug resistance. In the present study, molecular epidemiological analysis for antimalarial drug resistance genes in *Plasmodium falciparum* and *P. vivax* from the Mandalay region of Myanmar was performed. Blood samples were collected from patients infected with *P. falciparum* and *P. vivax* in four townships around the Mandalay region, Myanmar in 2015. Partial regions flanking major mutations in 11 antimalarial drug resistance genes, including seven genes (*pfdhfr*, *pfdhps*, *pfmdr-1*, *pfcrt*, *pfk13*, *pfubp-1*, and *pfcytb*) of *P. falciparum* and four genes (*pvdhfr*, *pvdhps*, *pvmdr-1*, and *pvk12*) of *P. vivax* were amplified, sequenced, and overall mutation patterns in these genes were analyzed. Substantial levels of mutations conferring antimalarial drug resistance were detected in both *P. falciparum* and *P. vivax* isolated in Mandalay region of Myanmar. Mutations associated with sulfadoxine-pyrimethamine resistance were found in *pfdhfr*, *pfdhps*, *pvdhfr*, and *pvdhps* of Myanmar *P. falciparum* and *P. vivax* with very high frequencies up to 90%. High or moderate levels of mutations were detected in genes such as *pfmdr-1*, *pfcrt*, and *pvmdr-1* associated with chloroquine resistance. Meanwhile, low frequency mutations or none were found in *pfk13*, *pfubp-1*, *pfcytb*, and *pvk12* of the parasites. Overall molecular profiles for antimalarial drug resistance genes in malaria parasites in the Mandalay region suggest that parasite populations in the region have substantial levels of mutations conferring antimalarial drug resistance. Continuous monitoring of mutations linked with antimalarial drug resistance is necessary to provide useful information for policymakers to plan for proper antimalarial drug regimens to control and eliminate malaria in the country.

## 1. Introduction

Malaria is an acute febrile infectious disease caused by *Plasmodium* species parasites transmitted by female *Anopheles* mosquitoes. Despite the remarkable decline of global malaria incidences in recent years, approximately 241 million people still had malaria and more than 620,000 died in 2020 globally [1]. For effective control and elimination of malaria, accurate diagnosis followed by proper treatment with antimalarial drugs is important [2]. However, global efforts toward controlling malaria have been challenged by the emergence and widespread of antimalarial drug resistance. Antimalarial drug resistance of malaria parasites is acquired by mutations or duplications in target genes, which can confer reduced drug susceptibility. Up to now, multiple genes associated with antimalarial drug resistances have been identified and major mutations inducing the resistances have been characterized [3,4,5]. The Great Mekong Subregion (GMS) has been recognized as a breeding hub for antimalarial drug resistant malaria parasites. Emergence and spreading of parasites resistant to antimalarial drugs threaten recent outstanding achievements in malaria control and challenge the goal for malaria elimination in the GMS [6]. Moreover, there are concerns on the emergence of artemisinin resistant parasites in the GMS [7,8,9,10,11].

Myanmar is a country where both *P. falciparum* and *P. vivax* are prevalent. It has the largest malaria burden in the GMS [12]. Similar to other countries in the GMS, parasites resistant to multiple antimalarial drugs have been reported in the country [13,14,15]. Currently, artemisinin-based combination therapy (ACT) and chloroquine (CQ) are applied as frontline treatment drugs for *P. falciparum* and *P. vivax*, respectively [12]. The unique geographical location of Myanmar, which connects GMS and South Asia, has also emphasized its importance as a bridge to spread antimalarial drug resistant parasites from GMS to South Asia countries [16]. Molecular analysis of antimalarial drug resistance markers has been validated as one effective tool for surveillance of resistance [3,4,5]. These markers serve as valuable molecular blueprints for mapping drug resistance status and planning malaria control measures [17]. Currently, several molecular studies reporting drug resistant parasites have performed in border areas of Myanmar [18,19,20,21,22,23,24,25,26,27,28], but it is lacked in the central Myanmar. Therefore, in the present study we investigated on the prevalence of resistant parasites in the Central region of Myanmar.

Using molecular profiling we report on the prevalence of resistant parasites in the central region, providing data that is important for the design of national malaria control strategy in Myanmar.

## 2. Materials and Methods

### 2.1. Blood Samples

**Figure 1:**
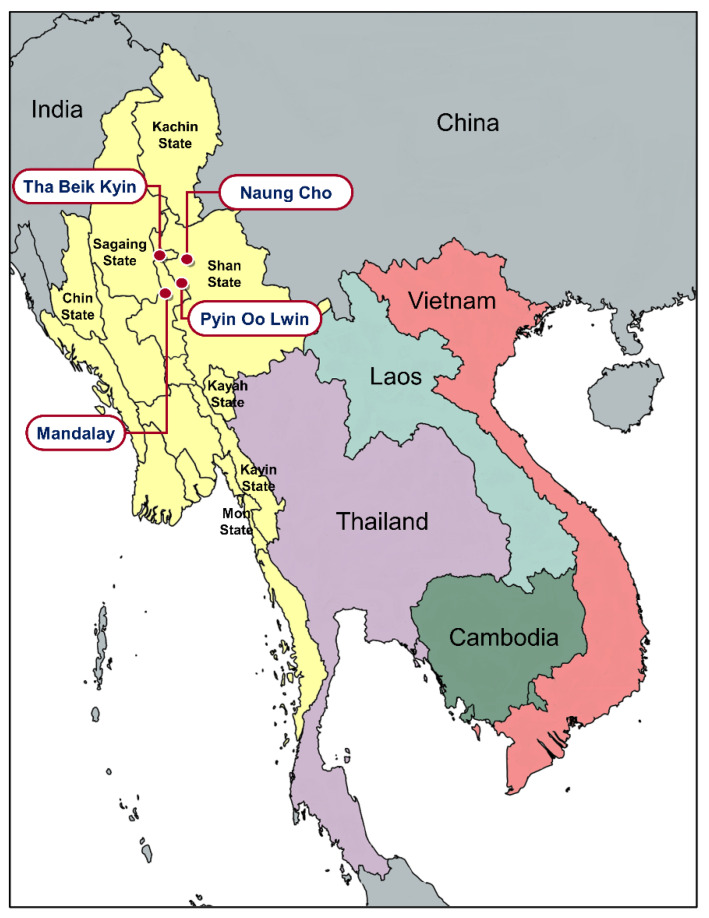
Blood samples collection site map. Blood samples of malaria patients were collected in the four townships, Mandalay region, Myanmar, in 2015.

Blood samples were collected from patients who were infected with *Plasmodium falciparum* and *P. vivax* in four townships, Mandalay, Tha Beik Kyin, Naung Cho, and Pyin Oo Lwin, around Mandalay region of Myanmar in 2015 [29] (Figure 1). The transmission intensity of malaria in the areas is low or hypo-endemic [1,20]. *Plasmodium* species were identified by microscopic examination of Giemsa stained thin and thick blood smears. Before antimalarial drug treatment, finger-prick blood samples were collected from the patients, spotted on Whatman 3 mm filter papers (GE Healthcare, Pittsburg, PA, USA), air-dried, and kept individually in sealed plastic bags at room temperature until further analysis. Informed consents were obtained from all patients before blood sampling. The study protocol was reviewed and approved by the Ethics Committee of the Ministry of Health, Myanmar (97/Ethics 2015) and the Biomedical Research Ethics Review Board of Inha University School of Medicine, Republic of Korea (INHA 15-013).

### 2.2. Amplification of Antimalarial Drug Resistance Genes

A total of 129 *P. falciparum* isolates (71 from Naung Cho, 6 from Pyin Oo Lwin, 20 from Mandalay, and 32 from Tha Beik Kyin) and 138 *P. vivax* isolates (112 from Naung Cho, 11 from Mandalay, and 15 from Tha Beik Kyin) were analyzed in this study. Parasite genomic DNA was extracted from the blood spots using the Blood DNA Kit (Qiagen, Hilden, Germany) according to the manufacturer’s protocols. Parasite species in the blood samples were further confirmed by species-specific nested polymerase chain reaction (PCR) for 18S ribosomal RNA (rRNA) gene [29,30]. The partial regions flanking major mutations associated with antimalarial drug resistance in *P. falciparum* and *P. vivax* marker genes were amplified by nested PCR, respectively. *P. falciparum* dihydrofolate reductase (*pfdhfr*), dihydropteroate synthase (*pfdhps*), multidrug resistance protein 1 (*pfmdr-1*), chloroquine resistance transporter (*pfcrt*), kelch propeller protein 13 (*pfk13*), cytochrome b (*pfcytb*), and ubiquitin carboxyl-terminal hydrolase 1 (*pfubp-1*) and *P. vivax* dihydrofolate reductase (*pvdhfr*), dihydropteroate synthase (*pvdhps*), multidrug resistance protein 1 (*pvmdr-1*), and kelch domain-containing protein (*pvk12*) were included. The primers used to amplify each drug resistance gene of *P. falciparum* and *P. vivax* were summarized in Table S1. Ex *Taq* DNA polymerase (Takara, Otsu, Japan) with proof-reading activity was used in all PCR steps to minimize the nucleotide misincorporation. The PCR products were analyzed on 1.5% agarose gel. Each PCR product was purified from the agarose gel and ligated into T&A cloning vector (Real Biotech Corporation, Banqiao City, Taiwan). Each ligation mixture was transformed into *Escherichia coli* DH5α competent cells and positive clones were selected by colony PCR using nested PCR primers for each gene. The plasmids were purified from the selected *E. coli* clones. The nucleotide sequences of the cloned gene fragments in the plasmids were analyzed by automatic Sanger sequencing method in both directions using M13 forward and M13 reverse primers. To verify the sequence accuracy, plasmids from at least three independent *E. coli* clones form each gene fragment in each isolate were sequenced. The nucleotide sequences of genes reported in this study have been deposited in the Genbank database following the accession numbers: *pfcrt* (OM981378–OM981466), *pfcytb* (OM981467–OM981568), *pfdhfr* (OM981569–OM981663), *pfdhps* (OM981664–OM981756), *pfk13* (OM981757–OM981831), *pfubp-1* (OM982024–OM982081), *pfmdr-1* (OM981832–OM981927, OM981928–OM982023), *pvmdr-1* (OM982314–OM982403), *pvdhfr* (OM982082–OM982174), *pvdhps* (OM982175–OM982252), and *pvk12* (OM982253–OM982313).

### 2.3. Sequence Polymorphism Analysis

The nucleotide and deduced amino acid sequences of each antimalarial drug resistance gene were annotated and analyzed with the Editseq, SeqMan, and Megalign programs in the DNASTAR package (DNASTAR, Madison, WI, USA). *P. falciparum* 3D7 *pfdhfr* (XM_001351443), HB3 *pfdhps* (PfHB3_080016200), 3D7 *pfmdr-1* (XM_001351751), 3D7 *pfcrt* (XM_001348968), 3D7 *pfk13* (PF3D7_1343700), 3D7 *pfcytb* (PF3D7_MIT02300), and 3D7 *pfubp-1* (PF3D7_0104300) were used as wild type reference sequences. For *P. vivax*, Sal I *pvdhfr* (PVX_089950), Sal I *pvdhps* (XM_001617159), Sal I *pvmdr-1* (AY618622), and Sal I *pvk12* (PVX_083080) were used as reference sequences for *P. vivax* genes.

### 2.4. Statistical Analysis

The comparison of haplotype frequency in each gene population between townships was performed to analyze precision and the extent of difference in the proportions. Chi-square test with 95% confidence intervals (CI 95%) and *p* value were calculated by using Graphpad Prism ver. 8.0.2 (San Diego, CA, USA).

## 3. Results

### 3.1. Molecular Profiles of Antimalarial Drug Resistance Genes in Myanmar P. falciparum

#### 3.1.1. Molecular Profiles of *pfdhfr* and *pfdhps*

**Figure 2:**
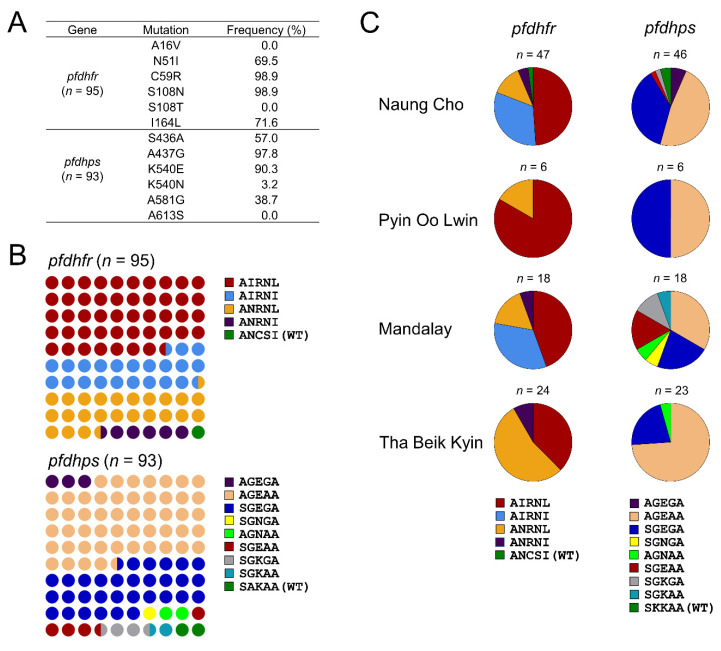
Frequencies and distributions of dihydrofolate reductase (*pfdhfr*) and dihydropteroate synthase (*pfdhps*) in Myanmar *P. falciparum* isolates. (**A**) Overall prevalence of mutations identified in *pfdhfr* and *pfdhps*. (**B**) Overall frequency of haplotypes of *pfdhfr* and *pfdhps*. The amino acid codons in haplotypes corresponded to the amino acids specified in (**A**). (**C**) Proportion of haplotypes of *pfdhfr* and *pfdhps* in each township.

The *pfdhfr* and *pfdhps* were successfully amplified from 95 and 93 Myanmar *P. falciparum* isolates, respectively. Both genes in the parasites showed high frequencies of mutations related to sulfadoxine-pyrimethamine (SP) resistance (Figure 2). For *pfdhfr*, C59R and S108N showed the highest frequencies of 98.9%, respectively, and followed by I164L (71.6%) and N51I (69.5%) (Figure 2A). These mutations were concurrent in the isolates, making diverse haplotypes. The quadruple mutation, AIRNL, was the most prevalent haplotype (45/95, 47.4%) followed by triple mutations, ANRNL (23/95, 24.2%) and AIRNI (21/95, 22.1%), and double mutation ANRNI (5/95, 5.3%) (Figure 2B). These mutant haplotypes were not evenly distributed in four townships. The frequency of each haplotype also differed by township (Figure 2C). The AIRNL showed the highest prevalence in Naung Cho (23/47, 48.9%), Pyin Oo Lwin (5/6, 83.3%), and Mandalay (8/18, 44.4%). However, ANRNL was more prevalent in Tha Beik Kyin (13/24, 54.2%). The AIRNI was detected only in Naung Cho (15/47, 31.9%) and Mandalay (6/18, 33.3%). For *pfdhps*, validated mutations associated with SP resistance were also detected with high frequencies: S436A (57.0%), A437G (97.8%), K540E (90.3%), K540N (3.2%), and A581G (38.7%) (Figure 2A). These mutations were also concurrent in isolates. Meanwhile, A613S was not detect in Myanmar *pfdhps*. Triple mutations AGEAA and SGEGA were predominant ones in Myanmar *pfdhps*, accounting for 51.6% (48/93) and 31.2% (29/93), respectively (Figure 2B). Mutant haplotypes including quadruple mutation (AGEGA), triple mutations (SGNGA and AGNAA), and double mutations (SGEAA, SGKGA, and SAKAA) were also identified, but their frequencies were low ranged from 1.1 to 4.3%. These mutant haplotypes were unevenly distributed in the four townships and significant difference in frequencies of the haplotypes were observed (χ^2^ = 22.76, *p* = 0.0016, CI 95%) (Table S2, Figure 2C). AGEAA and SGEGA showed high prevalence in all four townships, while AGEGA was found in only Naung Cho with a frequency of 6.5% (3/46). Diverse haplotypes were identified in Naung Cho and Mandalay, but not statistically different (χ^2^ = 34.55, *p* = 0.0754, CI 95%) (Table S2). However, Pyin Oo Lwin and Tha Beik Kyin showed simple haplotype compositions having only 2 or 3 distinct haplotypes. Synergistic effect of combined mutations in *pfdhfr* and *pfdhps* on SP resistance level has been classified previously: *pfdhfr* N51I/C59R/S108N + *pfdhps* A437G for partial resistance, *pfdhfr* N51I/C59R/S108N + *pfdhps* A437G/K540E for full resistance, and *pfdhfr* N51I/C59R/S108N + *pfdhps* A437G/K540E/A581G for super resistance [31]. Combined mutations in *pfdhfr* and *pfdhps* were identified in *P. falciparum* isolates only from Naung Cho and Mandalay, where more diverse haplotypes of mutations were detected. None of these combinations for partial resistance was found in Myanmar *P. falciparum* isolates. The combination considered as conferring full resistance was detected in three isolates (1 from Naung Cho and 2 from Mandalay). Super resistance was predicted for 8 isolates from Naung Cho and 1 isolate from Mandalay (Table 1). Besides these validated mutations for SP resistance, diverse minor mutations were also observed in Myanmar *pfdhps* and *pfdhfr* (Table S3).

#### 3.1.2. Molecular Profiles of *pfmdr-1* and *pfcrt*

**Figure 3:**
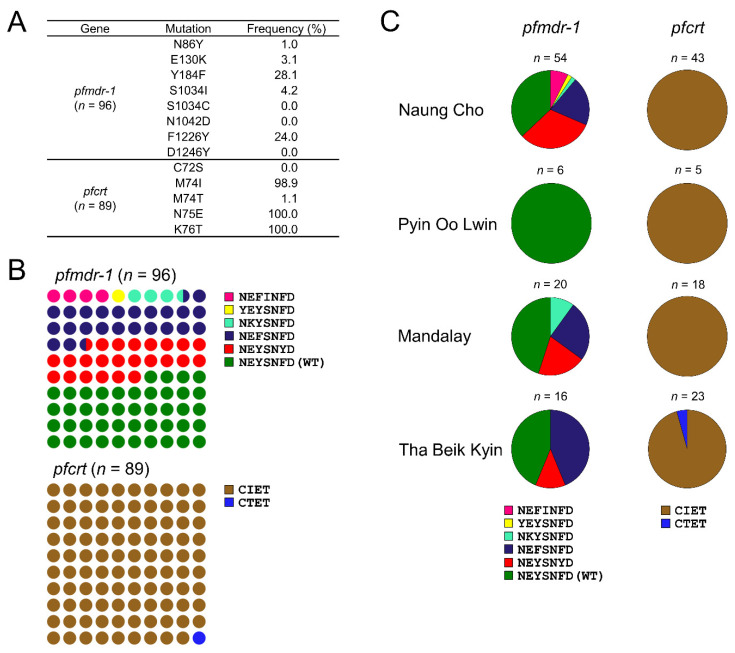
Frequencies and distributions of multidrug resistance 1 (*pfmdr-1*) and chloroquine resistance transporter (*pfcrt*) in Myanmar *P. falciparum* isolates. (**A**) Overall prevalence of mutations identified in *pfmdr-1* and *pfcrt*. (**B**) Overall frequency of haplotypes of *pfmdr-1* and *pfcrt*. The amino acid codons in haplotypes corresponded to the amino acids specified in (**A**). (**C**) Proportion of haplotypes of *pfmdr-1* and *pfcrt* in each township.

High frequency (56.2%) of mutations associated with CQ resistance was identified in 96 Myanmar *pfmdr-1*. Notable mutations found in Myanmar *pfmdr-1* were Y184F (28.1%) and F1226Y (24.0%) (Figure 3A). Although N86Y, E130K, and S1034I mutations were also observed, they showed lower frequencies ranging from 1.0% to 4.2%. NEFSNFD and NEYSNYD haplotypes were identified with the same frequency of 24.0% (Figure 3B). Frequencies of NEFINFD, YEYSNFD, and NKYSNFD were 4.2%, 1.0%, and 3.1%, respectively. Different *pfmdr-1* haplotypes were identified in *P. falciparum* from Naung Cho, Mandalay, and Tha Beik Kyin, but no mutant haplotype was identified in *P. falciparum* from Pyin Oo Lwin (χ^2^ = 20.86, *p* = 0.1414, CI 95%) (Figure 3C, Table S2). In the case of *pfcrt*, very high levels of mutations linked to CQ resistance were found. All Myanmar *pfcrt* had major mutations of M74I/T, N75E, and K76T known to closely associated with CQ resistance. Frequencies of M74I, M74T, N75E, and K76T were 98.9%, 1.1%, 100%, and 100%, respectively (Figure 3A). These mutations resulted in two quadruple mutation haplotypes of CIET and CTET, with CIET being highly prevalent (88/89, 98.9%) (Figure 3B). CIET was found in all Myanmar *P. falciparum* isolates except for one isolate from Tha Beik Kyin, which had the CTET haplotype (Figure 3C). Combination of mutations in the two genes, *pfmdr-1* N86Y + *pfcrt* K76T suspected to be associated with amodiaquine (AQ) and CQ resistance [32], was identified in one isolate from Naung Cho (Table 1). Besides these validated or associated mutations, a number of minor mutations were also found in *pfmdr-1* and *pfcrt* of Myanmar *P. falciparum* (Table S3).

#### 3.1.3. Molecular Profiles of *pfk13*, *pfubp-1*, and *pfcytb*

**Figure 4:**
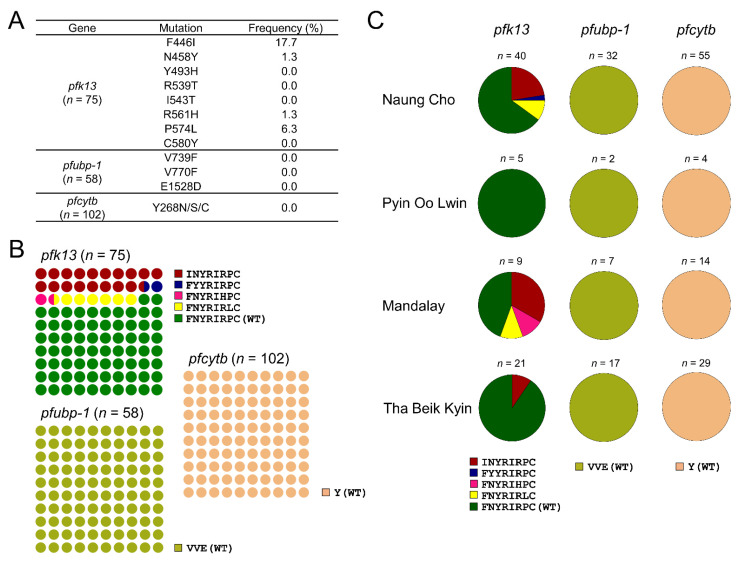
Frequencies and distributions of Kelch 13 (*pfk13*), ubiquitin specific protease 1 (*pfubp-1*) and cytochrome b (*pfcytb*) in Myanmar *P. falciparum* isolates. (**A**) Overall prevalence of mutations identified in *pfk13*, *pfubp-1*, and *pfcytb*. (**B**) Overall frequency of haplotypes of *pfk13*, *pfubp-1*, and *pfcytb*. The amino acid codons in haplotypes corresponded to the amino acids specified in (**A**). (**C**) Proportion of haplotypes of *pfk13*, *pfubp-1*, and *pfcytb* in each township.

A total of 75 sequences of *pfk13* were obtained from Myanmar *P. falciparum* isolates. Although wild type *pfk13* was prevalent (54/75, 72.0%), four mutations (F446I, N458Y, R561H, and P574L) associated with artemisinin resistance were identified. F446I was the most common mutation accounting for 17.7%, followed by P574L with a frequency of 6.7% (Figure 4A). INYRIRPC was the most prevalent mutant haplotype with a frequency of 18.7%, followed by FNYRIRLC (6.7%) (Figure 4B). Two mutant haplotypes, FYYRIRPC and FNYRIHPC, accounted for 1.3%, respectively. These mutant haplotypes were detected in the isolates from Naung Cho, Mandalay, and Tha Beik Kyin, but not in isolates from Pyin Oo Lwin (χ^2^ = 16.86, *p* = 0.1549, CI 95%) (Table S2). Haplotype diversity was greater in Naung Cho and Mandalay (Figure 4C). Sequence analyses of 102 *pfcytb* and 58 *pfubp-1* sequences revealed no mutation associated with drug resistance (V739F, V770F, or E1528D in *pfubp-1* or Y268N/S/C in *pfcytb*) in Myanmar isolates. Beyond these validated mutations, minor mutations were identified in *pfk13*, *pfubp-1*, and *pfcytb* of Myanmar *P. falciparum* (Table S3).

#### 3.1.4. Summary of Mutations in Multiple Antimalarial Drug Resistance Genes

**Figure 5:**
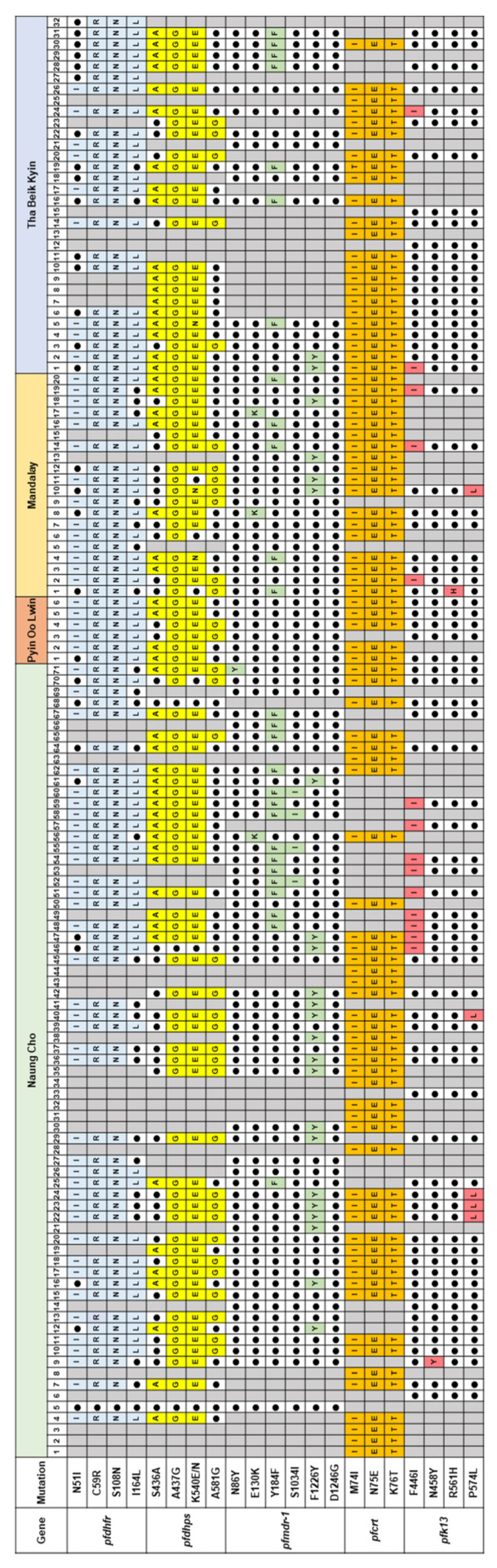
Combinational analysis of the mutations in five genes associated with antimalarial drug resistance in *P. falciparum*. Mutations in each gene highlighted with different colors and wild type residues shown as closed circles. Grey boxes represented samples failed to amplify.

Although not all antimalarial drug resistance genes were successfully amplified in all Myanmar *P. falciparum* isolates, comparative analysis of molecular profiles of the five genes except *pfubp-1* and *pfcytb* in each isolate suggested a high prevalence of multiple drug resistance of the parasite, implying resistance against at least two different antimalarial drugs (Figure 5). Among the 129 *P. falciparum* isolates analyzed, 9 shared validated mutations in *pfdhfr*, *pfdhps*, *pfmdr-1*, *pfcrt*, and *pfk13*, suggesting their potent resistance against multiple antimalarial drugs including SP, CQ, and artemisinin. Concurrent mutations in genes associated with SP and CQ resistance were also identified in 85 Myanmar *P. falciparum* isolates.

### 3.2. Molecular Profiles of Antimalarial Drug Resistance Genes in Myanmar P. vivax

#### 3.2.1. Molecular Profiles of *pvdhfr* and *pvdhps*

**Figure 6:**
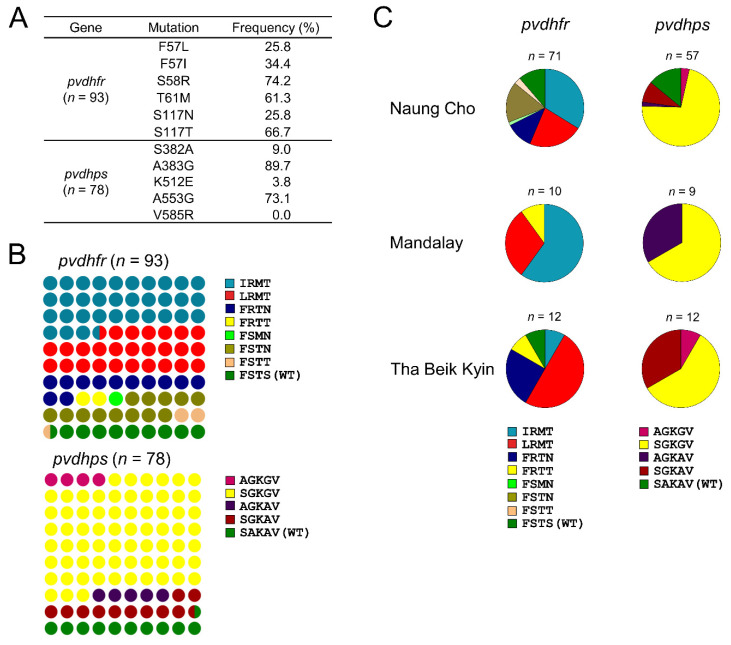
Frequencies and distributions of dihydrofolate reductase (*pvdhfr*) and dihydropteroate synthase (*pvdhps*) in Myanmar *P. vivax* isolates. (**A**) Overall prevalence of mutations identified in *pvdhfr* and *pvdhps*. (**B**) Overall frequency of haplotypes of *pvdhfr* and *pvdhps*. The amino acid codons in haplotypes corresponded to the amino acids specified in (**A**). (**C**) Proportion of haplotypes of *pvdhfr* and *pvdhps* in each township.

The *pvdhfr* was successfully amplified from 93 Myanmar *P. vivax* isolates. The majority of these isolates carried mutations related to SP resistance. F57L/I, S58R, T61M, and S117N/T were found in 60.2%, 74.2%, 61.3%, and 92.5% of samples, respectively (Figure 6A). A total of eight distinct haplotypes of *pvdhfr* having quadruple, double, or single mutation were observed in Myanmar *pvdhfr*, with IRMT being the most prevalent (31/93, 33.3%), followed by LRMT (25/93, 26.9%) (Figure 6B). Frequencies of FSTN, FRTN, and wild type FSTS were 12.9%, 11.8%, and 9.7%, respectively. Meanwhile, frequencies of FRTT, and FSTT, and FSMN were low, ranging from 1.1% to 2.2%. Distributions of these mutant haplotypes were differed by township, but not significantly different (χ^2^ = 22.62, *p* = 0.0068, CI 95%) (Table S2). The IRMT was prevalent in Naung Cho and Mandalay, whereas LRMT was predominant in Tha Beik Kyin (Figure 6C). Greater haplotype diversity was found in *P. vivax* isolates from Naung Cho and Tha Beik Kyin than those from Mandalay. For *pvdhps*, S382A, A383G, K512E, and A553G were detected in 78 Myanmar *P. vivax* isolates with frequencies of 9.0%, 89.7%, 3.8%, and 73.1%, respectively (Figure 6A). There was no V585R detected in Myanmar *pvdhps*. These mutations generated five different haplotypes of Myanmar *pvdhps* harboring triple mutations (AGKGV), double mutations (SGKGV and AGKAV), and single mutation (SGKAV) (Figure 6B). The SGKGV was the most prevalent haplotype, having a frequency of 69.2%. Frequencies of SGKAV, AGKAV, and AGKGV were 11.5%, 5.1%, and 3.9 %, respectively. Meanwhile, the frequency of SAKAV (wild type) was 10.3%. The SGKGV was a prevalent haplotype in all three townships (Figure 6C). Similar to *pvdhfr*, greater haplotype diversity of *pvdhps* was detected in *P. vivax* isolates from Naung Cho and Tha Beik Kyin than from Mandalay (χ^2^ = 26.41, *p* = 0.0009, CI 95%) (Table S2). Allele combination between *pvdhfr* and *pvdhps* harboring multiple mutations in these two genes is known to contribute to synergistic SP resistance [33]. Sixty-three Myanmar *P. vivax* isolates carried concurrent mutations in both *pvdhfr* and *pvdhps* (Table 2). Besides these major mutations, diverse minor mutations were also found in the genes (Table S4).

#### 3.2.2. Molecular Profiles of *pvmdr-1* and *pvk12*

**Figure 7:**
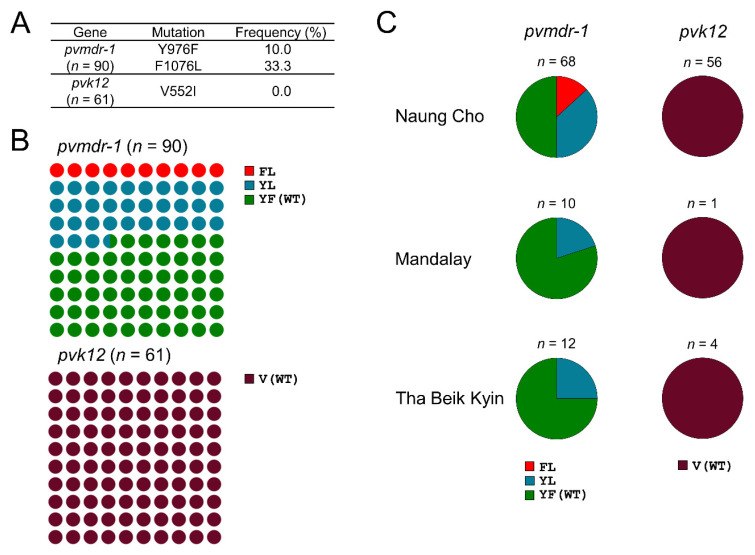
Frequencies and distributions of multidrug resistance 1 (*pvmdr-1*) and kelch 12 (*pvk12*) in Myanmar *P. vivax* isolates. (**A**) Overall prevalence of mutations identified in *pvmdr-1* and *pvk12*. (**B**) Overall frequency of haplotypes of *pvmdr-1* and *pvk12*. The amino acid codons in haplotypes corresponded to the amino acids specified in (**A**). (**C**) Proportion of haplotypes of *pvmdr-1* and *pvk12* in each township.

Moderate levels of mutations associated with CQ resistance were detected in *pvmdr-1* of Myanmar *P. vivax* isolates. Two mutations, Y976F and F1076L, were identified in Myanmar *pvmdr-1* with frequencies of 10.0% and 33.3%, respectively (Figure 7A). Frequencies of FL haplotype and YL haplotype were 10.0% and 33.3%, respectively (Figure 7B). FL was detected only in Naung Cho isolates, while YL was commonly identified in all isolates from Naung Cho, Mandalay, and Tha Beik Kyin (Figure 7C). Meanwhile, V552I, which was suspected to be associated with artemisinin resistance, was not detected in Myanmar *pvk12*. Diverse minor mutations were also observed in Myanmar *pvmdr-1* and *pvk12* (Table S4).

#### 3.2.3. Summary of Mutations in Multiple Antimalarial Drug Resistance Genes

**Figure 8:**
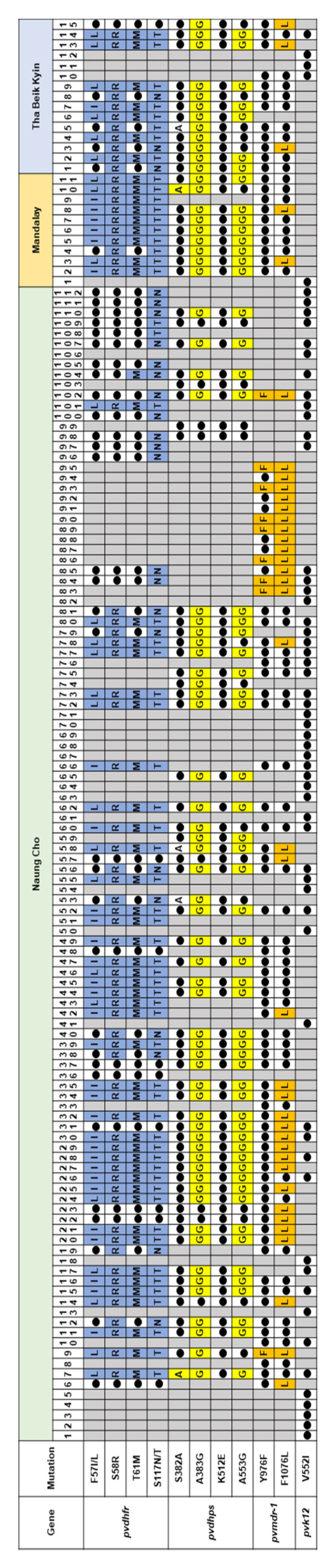
Combinational analysis of the mutations in four genes associated with antimalarial drug resistance in *P. vivax*. Mutations in each gene highlighted with different colors and wild type residues shown as closed circles. Grey boxes represented samples failed to amplify.

Consistent with *P. falciparum*, not all antimalarial drug resistance genes were successfully amplified in all Myanmar *P. vivax* isolates analyzed. However, comparative analysis of molecular profiles of the four genes in each *P. vivax* isolate implied substantial levels of multiple drug resistances in Myanmar isolates (Figure 8). Among the 138 *P. vivax* isolates analyzed, 21 had concurrent mutations in *pvdhfr*, *pvdhps*, and *pvmdr-1*, suggesting their potent roles in SP and CQ resistance.

## 4. Discussion

Antifolate drugs represented by SP have been extensively deployed in Myanmar in the past decades. However, they have been withdrawn due to widespread use of resistant *Plasmodium* species against these drugs. As expected, extremely high levels of mutations strongly associated with SP resistance were detected in both *P. falciparum* and *P. vivax* analyzed in this study. *P. falciparum* isolates from Mandalay region had major mutations that were strongly associated with SP resistance in *pfdhfr* and *pfdhps* with high frequencies. In the case of *pfdhfr*, these mutations were commonly identified as concurrent mutations rather single mutations in the gene. Frequencies of quadruple mutation (AIRNL), triple mutation (AIRNI or ANRNL), and double mutation (ANRNI) were comparable to or slightly different from those of *P. falciparum* collected from northern (Banmauk, Sagaing State and Laiza, Kachin State) and western (Paletwa, Chin State) Myanmar [25,34]. Concurrent mutations were also identified in *pfdhps*. Frequencies of the mutations were similar to or slightly higher than those of *P. falciparum* collected from northern (Banmauk, Sagaing State and Laiza, Kachin State) and western (Paletwa, Chin State) Myanmar [25,34]. However, more diverse haplotypes of *pfdhps* were identified in *P. falciparum* isolates collected from Mandalay region. Combined mutations in *pfdhfr* and *pfdhps* have been recognized as reliable predictors for SP treatment failure [31,35]. These mutations were also detected in Myanmar isolates analyzed in this study. Similar proportions of these combinations have been reported in northern Myanmar, Thailand and Cambodia [25,36]. High rates of *pvdhps* and *pvdhfr* mutations in Myanmar *P. vivax* have been reported previously [17,19,25]. High levels of mutations associated with SP resistance were also observed in *pvdhps* and *pvdhfr* in *P. vivax* isolates from Mandalay region, which were comparable to parasites from southern (Kayah, Mon, and Kayin), northern (Laiza, Kachin State), and western (Sagaing and Buthidaung) Myanmar [19,25]. These molecular profiles for SP-resistant *Plasmodium* population in Myanmar should be underscored in that mutations in *dhfr* and *dhps* conferring SP resistance to both *P. falciparum* and *P. vivax* were still highly preserved in the parasite population of Myanmar, albeit antifolate drugs were not used for malaria treatment in recent few decades. The reason for why selective pressure still acting to maintain *dhfr* and *dhps* mutations for SP resistance in Myanmar *Plasmodium* population is currently unclear. Sulfa based drugs such as cotrimoxazole, sulfamethoxazole and trimethoprim used to treat bacterial infections can be one cause. Further comprehensive studies are needed to elucidate it.

CQ had been largely applied in Myanmar to treat both falciparum malaria and vivax malaria in the past. However, CQ resistant *P. falciparum* began to be reported in Myanmar in 1970s and has rapidly spread throughout the country [37]. Mutations in *pfcrt* are primary indicators for CQ resistance, especially K76T mutation [38,39]. They can also influence susceptibility to quinine (QN), mefloquine (MQ), halofantrine (HF), and amodiaquine (AQ) [40]. High levels of mutations including M74I, N75E, and K76T were detected in Myanmar *pfcrt* analyzed in this study. Moreover, all *P. falciparum* isolates harbored the triple mutation, CIET or CTET. These values were greater than those of *P. falciparum* isolates collected from northern (Laiza and Banmauk; 76.5%) and western (Paletwa; 95.8%) Myanmar [25]. Meanwhile, frequencies of two validated major mutations linked to CQ resistance in *pfmdr-1*, Y184F and F1226Y, were relatively low in samples analyzed in this study. Concurrent mutations in *pfcrt* (K67T) and *pfmdr-1* (N86Y, Y184F, S1034I, N1042D, and D1246Y) are known to increase CQ resistance [41]. Combination of *pfcrt* K76T and *pfmdr-1* N86Y was identified in only one isolate from Naung Cho, while combined mutations of *pfcrt* K76T and *pfmdr-1* Y184F were detected with a frequency of 22.5%, which was lower than that in *P. falciparum* isolates collected from northern Myanmar (Laiza and Banmauk: 76.4%), but higher than that in *P. falciparum* isolates collected from western Myanmar (Paletwa: 12.5%) [25]. The N86Y/Y184F mutation in *pfmdr-1* also has proposed to decrease parasite susceptibility to lumefantrine and MQ by enhancing digestive vacuole transport efficacy [42]. This double mutation was not identified in *pfmdr-1* in the parasites analyzed in this study, suggesting lumefantrine and MQ may still applicable as partner drugs for ACT in Mandalay region. These results collectively indicate that substantial levels of CQ-resistant *P. falciparum* are prevalent in Myanmar, although the drug was withdrawn from the country in the 1970’s for radical cure of falciparum malaria. Nonetheless, CQ is still continuously used as a frontline treatment drug for vivax malaria in Myanmar. This might have contributed to a stable maintenance of *pfcrt* mutations in Myanmar *P. falciparum* population. Although CQ is still the drug of choice for vivax malaria in Myanmar, CQ-resistant *P. vivax* was first detected in Myanmar in the 1990s [37]. Declined therapeutic responses of *P. vivax* to CQ have been recently reported in the China-Myanmar border and southern Myanmar [21,43,44,45,46]. Although controversial as molecular markers for CQ resistance in *P. vivax* still remains, *pvmdr-1* and *pvcrt-o* have been queried since these two genes are orthologues of *pfmdr-1* and *pfcrt*, which are validated molecular markers for CQ resistance in *P. falciparum* [3,47]. The Y967F in *pvmdr-1* is a major mutation that can decrease in vitro sensitivity of CQ in *P. vivax*. Double mutation of Y976F/F1076L in *pvmdr-1* can significantly increase CQ resistance [48,49]. The frequency of FL double mutation in this study was 11.8%. Different levels of these mutations were reported in *P. vivax* isolates from other regions of Myanmar, including Y976F in southern (Kawthaung and Shwegyin, 20.9%), northern (Laiza, 3.8%), and western (Buthidaung, 1.7%) and F1076L in western (Buthidaung; 63.3%), southern (Kawthaung and Shwegyin; 45.9%), and northern (Laiza, 78.8%) [20,50]. These findings imply that Myanmar *P. vivax* population also has molecular profiles for potent CQ resistance, albeit frequencies of these mutations differed by region.

Myanmar occupies an important geographical location in containment of artemisinin-resistant parasites, as the country has the largest malaria burden in GMS and bridges GMS and South Asia. Furthermore, artemisinin resistance risk areas are challenging for malaria control due to high rates of migration at border areas, remote forested and mountainous areas, and reliance on private health care providers [51]. ACT regimen has changed several times given the selection of resistance markers to the partner drugs. ACT (artemether–lumefantrine [AL], dihydroartemisinin–piperaquine [DHA-PPQ] or artesunate-mefloquine [AS-MQ]) has been presently adopted as the frontline treatment for uncomplicated falciparum malaria [1,52]. In Mandalay region, the first line ACT for uncomplicated *P. falciparum* is AL and the alternative ACT is AS-MQ [53]. Delayed parasite clearance against artemisinin has been recognized to be associated with mutations in *pfk13*. Nine mutations (F446I, N458Y, M476I, Y493H, R539T, I543T, P553L, R561H, and C580Y) in *pfk13* are validated or associated with in vivo and in vitro ACT resistance in *P. falciparum* [6]. Of these 9 mutations, 4 mutations (F446I, N458Y, R561H, and P574L) were found in *pfk13* of *P. falciparum* isolates analyzed in this study, with F446I showing the highest prevalence of 17.7%. This value was lower than those of previously reported *P. falciparum* isolates from Myitkyina (41.9%) and China-Myanmar border (55.9%), but higher than those from Tha Beik Kyin (15.5%) and southern Myanmar including Myanmar-Thailand border (less than 10%) [15,22,25,54]. The P574L (6.3%) was also detected with comparable or slightly lower frequency compared to a previous report on isolates collected from the Myanmar-China border [55]. Interestingly, C580Y, the key mutation of artemisinin resistance, was not identified in *P. falciparum* isolates analyzed in this study. This mutation has been reported in limited areas of Myanmar, southern (Myanmar-Thailand border) and northern (Kyauk Mee, Shan State) with a frequency of 11.4% linked to other two *pfk13* mutations, R561H and F446I [20,56]. Several therapeutic efficacy studies on artemisinin-based combinations such as AL, AS-MQ, and DHA-PPQ for the treatment of uncomplicated *P. falciparum* in Myanmar have been performed [34,54,55,56]. Efficacy of these antimalarial drugs is likely to remain high in Myanmar albeit *pfk13* mutations were reported in some regions [55,56], suggesting ACT is still effective in the country. In addition, pyronaridine-artesunate also displayed high efficacy for both uncomplicated *P. falciparum* and *P. vivax* malaria, implying it can be included in the national malaria treatment protocols of Myanmar [56]. Molecular mechanism for artemisinin resistance of *P. vivax* has not been clearly elucidated yet. However, V552I mutation in *pvk12*, an orthologous of *pfk13*, was supposed to be associated with artemisinin resistance of the parasite [57]. In areas where *P. falciparum* and *P. vivax* are co-endemic, these two species can share the same mosquito vectors and human hosts and they are often subject to similar forces of natural selection [2,58]. Therefore, wide employment of ACT to cure *P. falciparum* infections might exert collateral selective pressure to *P. vivax* populations. Although *P. vivax* might have been exposed to higher selective pressure by artemisinin for the last few decades after ACT was introduced, V552I was not detected in *pvk12* of any Myanmar *P. vivax* isolates analyzed, albeit 48 other minor mutations were discovered. V552I was not reported in Cambodia or Myanmar *P. vivax* population either [26,57,59,60,61]. Considering limited knowledge on *pvk12* and mutations associated with artemisinin resistance in the gene, further study is needed to determine the role of these mutations with artemisinin resistance.

Mutations in *pfubp-1* is also known to contribute to artemisinin resistance of *P. falciparum* [62]. Especially, mutations at D1525E and E1528D in *pfubp-1* are likely to be closely associated with delayed parasite clearance [63]. These mutations were not observed in *pfubp-1* of *P. falciparum* analyzed in this study. Although a few non-synonymous mutations including H1459R, W1470C, D1522G, N1548D, and H1550R/L were detected with low frequencies, it is currently unclear whether these mutations could confer artemisinin resistance in *P. falciparum*. This needs to be investigated further. Mutations in *pfcytb* of *P. falciparum* can induce treatment failures of atovaquone against *P. falciparum* by inhibiting parasite mitochondria electron transport mechanism [64]. A previous study has suggested that Y268N/S/C in *pfcytb* is related to resistance of atovaquone-proguanil (Malarone) in *P. falciparum* [65]. This mutation was not detected in *P. falciparum* isolates analyzed in this study, coinciding with a previous study on Myanmar *P. falciparum* isolates [66]. However, continuous monitoring this mutation in Myanmar *P. falciparum* should be necessary as Malarone is a drug that has widely used for chemoprophylactic purposes, especially for travelers.

Overall molecular profiles of multiple antimalarial drug resistance genes in Myanmar *P. falciparum* and *P. vivax* populations suggest mild geographical heterogeneity in Myanmar. However, overall rates of mutations validated or associated with SP and CQ resistances are likely to be maintained at high or substantial levels. Meanwhile, mutations conferring ACT-resistance remained at relatively low levels. Non-neglected levels of parasite population carrying multiple drug resistance characters were also found. Increasing genetic diversity of *P. falciparum* and *P. vivax*, albeit marked reduction of recent malaria incidences, raises a concern for dynamic changes of genetic structure and expansion of genetic heterogeneity of *Plasmodium* population in Myanmar [67,68]. Increasing movement of human populations may facilitate changes of parasite transmission patterns and spreading of drug-resistant *Plasmodium* populations in the country. Asymptomatic cases are also a great concern since asymptomatic patients serve as silent reservoirs to continue transmission of malaria and antimalarial drug resistant parasites [23,26,29]. Similar to other countries in the GMS, *P. vivax* is becoming a predominant species in Myanmar with recent decrease of falciparum malaria cases. Mixed infections are also found frequently [29,69]. Continuous molecular surveillance for antimalarial drug resistance in *Plasmodium* parasites nationwide would be necessary to update and reset guidance for the use of antimalarial drugs in Myanmar.

## 5. Conclusions

High or substantial levels of mutations in antimalarial drug resistance genes were detected in both *P. falciparum* and *P. vivax* isolates collected from the Mandalay region, central Myanmar. Non-neglectable proportions of combined mutations in SP- and CQ-resistance genes in parasite populations suggest that multiple drug resistance parasites are prevalent in Mandalay region. However, mutations in *pfk13*, *pfubp-1*, and *pvk12* were identified at low frequency or were absent, suggesting that artemisinin resistance might not be a great concern, at least in the Mandalay region of Myanmar in 2015. But further assessment of artemisinin resistance in larger numbers of samples would be necessary to trace the current change in the frequencies of the mutations in the region. No mutation in *pfcytb* also suggests that atovaquone may be effective against Myanmar *P. falciparum* so far. Frequency of mutations in the genes analyzed in this study differed slightly by township. It can be caused by the different size of samples in each township. Considering that these two parasites, *P. falciparum* and *P. vivax*, share the same vectors and human hosts and that they are affected by collateral selective pressure of antimalarial drugs, continuous monitoring of antimalarial drug resistances in both parasites is necessary. The limitations of this study are the small sample size per region and time of sampling, which limits the applicability and generalizability of the study findings to contemporary surveillance programs at current. However, similar antimalarial drug resistance profiles in the central region of Myanmar with border areas of the country emphasize the necessity of continuous monitoring in the larger areas to provide useful information for policy makers to design proper antimalarial drug strategy for effective control and elimination of malaria in Myanmar.

## Figures and Tables

**Table 1:** Combination of mutations between genes related with multidrug resistance and resistance level of Myanmar *P. falciparum*.

**Gene** **Combination**	**Mutations**	**Naung Cho** **(*n* = 38)**	**Mandalay** **(*n* = 17)**	**Pyin Oo Lwin (*n* = 6)**	**Tha Beik Kyin (*n* = 19)**
*pfdhfr* + *pfdhps*	N51I/C59R/S108N + A437G	0	0	0	0
	N51I/C59R/S108N + A437G/K540E	1 (2.6%)	2 (11.8%)	0	0
	N51I/C59R/S108N + A437G/K540E/A581G	8 (21.1%)	1 (5.9%)	0	0
**Gene** **combination**	**Mutations**	**Naung Cho ** **(*n* = 16)**	**Mandalay ** **(*n* = 11)**	**Pyin Oo Lwin ** **(*n* = 0)**	**Tha Beik Kyin ** **(*n* = 6)**
*pfmdr-1* + *pfcrt*	N86Y + K76T	1 (6.3%)	0	0	0

*n*: number of samples amplified *pfdhfr* + *pfdhps* or *pfmdr-1* + *pfcrt.*

**Table 2:** Combination of mutations between genes related with multidrug resistance and resistant level of Myanmar *P. vivax*.

Gene Combination	Mutations	Naung Cho (*n* = 43)	Mandalay (*n* = 9)	Tha Beik Kyin (*n* = 12)
*pvdhfr* + *pvdhps*	F57I/S58R/T61M/S117T + S382A/A383G/A553G	1 (2.3%)	0	0
	F57I/S58R/T61M/S117T + A383G/A553G	19 (41.1%)	5 (55.6%)	1 (8.3%)
	F57L/S58R/T61M/S117T + A383G/A553G	7 (16.3%)	2 (22.2%)	5 (41.7%)
	F57I/S58R/T61M/S117T + S382A/A383G	0	0	0
	F57L/S58R/T61M/S117T + S382A/A383G	0	1 (11.1%)	0
	F57L/S58R/T61M/S117T + A383G	2 (4.7%)	0	1 (8.3%)
	F57I/S58R/T61M/S117T + A383G	1 (2.3%)	0	0
	S58R/S117N + A383G/A553G	6 (13.9%)	0	1 (8.3%)
	S58R/S117T + S382A/A383G/A553G	0	0	1 (8.3%)
	S58R/S117N + S382A/A553G	1 (2.3%)	0	0
	T61M/S117N + A383G/A553G	1 (2.3%)	0	0
	S58R/S117T + A383G/A553G	0	1 (11.1%)	0
	S117N + A383G/A553G	3 (7.0%)	0	0
	S58R/S117N + A553G	0	0	2 (16.7%)

*n*: number of samples amplified *pvdhfr* + *pvdhps.*

## Data Availability

The data supporting the conclusions of this article are provided within the article. The original datasets analyzed in this study are available from the corresponding author upon request. The nucleotide sequences reported in this study have been deposited in the GenBank database under the accession numbers: *pfcrt* (OM981378–OM981466), *pfcytb* (OM981467–OM981568), *pfdhfr* (OM981569–OM981663), *pfdhps* (OM981664–OM981756), *pfk13* (OM981757–OM981831), *pfubp-1* (OM982024–OM982081), *pfmdr-1* (OM981832–OM981927, OM981928–OM982023), *pvmdr-1* (OM982314–OM982403), *pvdhfr* (OM982082–OM982174), *pvdhps* (OM982175–OM982252), and *pvk12* (OM982253–OM982313).

## Supplementary Materials

The following supporting information can be downloaded at: https://www.mdpi.com/article/10.3390/microorganisms10102021/s1, Table S1: PCR primers to amplify *P. falciparum* and *P. vivax* drug resistance genes; Table S2: The distribution of mutant haplotypes of antimalarial drug resistance between each township in Myanmar *P. falciparum* and *P. vivax* isolates; Table S3: Minor mutations identified in drug resistance genes of Myanmar *P. falciparum*; Table S4: Minor mutations identified in drug resistance genes of Myanmar *P. vivax* [70,71,72,73,74,75].
