# Supplementary material for: Molecular Profiles of Multiple Antimalarial Drug Resistance Markers in Plasmodium falciparum and Plasmodium vivax in the Mandalay Region, Myanmar
Source: Microorganisms. 2022 Oct 13;10(10):2021. doi: 10.3390/microorganisms10102021 (PMC9612053; doi:10.3390/microorganisms10102021)
Supplement: Supplementary file 1 [file microorganisms-10-02021-s001.zip › Supplement file 1_Table S1.pdf]

Table S1: PCR primers to amplify *P. falciparum* and *P. vivax* drug resistance genes

PCR primers to amplify *P. falciparum drug* resistance genes

Gene	Primer sequences (5´to 3´)	Approximate size (bp)	Coverage mutations	References
pfdhfr	Forward: TTTATGATGGAACAAGTCTGC<br>Reverse: CTAGTATATACATCGCTAACA<br>Nested forward: TGATGGAACAAGTCTGCGACGTT<br>Nested reverse: CTGGAAAAAATACATCACATTCATATG	594	A16V, N51L, C59R, S108N/T, I194L	(Pearce et al., 2003)
pfdhps	Forward: GATTCTTTTTCAGATGGAGG<br>Reverse: TTCCTCATGTAATTCATCTGA<br>Nested forward: AACCTAAACGTGCTGTTCAA<br>Nested reverse: AATTGTGTGATTTGTCCACAA	711	S436A, A437G, K540E/N, A518G, A613S	(Pearce et al., 2003)
pfcrt	Forward: TGGCTCACGTTTAGGTGGAG<br>Reverse: TGTTACAAAACTATAGTTACC<br>Nested forward: TGTGCTCATGTGTTTAAACTT<br>Nested reverse: CAAAACTATAGTTACCAATTTTG	145	C72S, M74I/T, N75E, K76T	(Naoshima-Ishibashi et al., 2007)*
pfcytb	Forward: CTCTATTAATTTAGTTAAAGCACA<br>Reverse: ACAGAATAATCTCTAGCACC<br>Nested forward: TTAAAGCACACTTAATAAATTACC<br>Nested reverse: AGCACCAAAAATCATTTTAAATTG	911	Y268N/S/C	(Schwöbel et al., 2003)
pfubp-1	Forward: CGCCCGTACTATGAAGAAGATC<br>Reverse: GGCTTTTACCTGAACTGTTCAGG<br>Nested forward: CGTAAACAGAATATTCAGGATTGC<br>Nested reverse: CTAGCCCTTTATTATCATTATCG	304	V739F, V770F, E1528D	(Yan et al., 2020)
pfk13	Forward: CGGAGTGACCAAATCTGGGA<br>Reverse: GGGAATCTGGTGGTAACAGC<br>Nested forward: GCCAAGCTGCCATTCATTTG<br>Nested reverse: GCCTTGTTGAAAGAAGCAGA	849	F446I, N458Y, Y493H, R539T, I543T, R561H, P574L, C580Y	(Huang et al., 2015)
pfmdr-1 (A)	Forward: TGAAAGATGGGTAAAGAGCAGA<br>Reverse: CCATACCAAAAACCGAATGC<br>Nested forward: TGAACAAAAAGAGTACGGCTGA<br>Nested reverse: AAATTAACGGAAAAACGCAAG	549	N86Y, E130K, Y184F	In this study
pfmdr-1 (B)	Forward: CAAAAGTAAAGAAATTGAGAAAA<br>Reverse: AAACTTACTAACACGTTTAACATC<br>Nested forward: CAAGCGGAGTTTTTGCATTT<br>Nested reverse: CAATGTTGCATCTTCTCTTCCA	938	S1034I,/C, N1042D, F1226Y, D1246Y	In this study

PCR primers to amplify *P. vivax* drug resistance genes

Gene	Primer sequences (5´to 3´)	Approximate size (bp)	Coverage mutations	References
pvmdr-1	Forward: GGATAGTCATGCCCCAGGATTG<br>Reverse: CATCAACTTCCCGGCGTAGC<br>Nested forward: TGCTGTCAGCACATATTAACAG<br>Nested reverse: TAGCTTCCCGTAAATAAAAAGG	568	Y976F, F1076L	(Chung et al., 2015)<br><br>In this study
pvdhfr	Forward: ATGGAGGACCTTTCAGATGTATTTGACATT<br>Reverse: TTAGGCGGCCATCTCCATGGTTATTTTATC<br>Nested forward: TTCAGATGTATTTGACATTTACGCCA<br>Nested reverse: TTGCTGTAAACCAAAAAGTCCAGAGT	701	F57L/I, S58R, T61M, S117N/T	In this study
pvdhps	Forward: AAGAATAATAAGAGGCTATACGTATTG<br>Reverse: AAATCAGAGAAAGGACGAATTCCTACC<br>Nested forward: CTATACGTATTGAAAGATAAAGTGTC<br>Nested reverse: CGAATTCCTACCGCAAATATTTTTCTG	1092	S382A, A383G, K512E, A553G, V585R	In this study
pvk12	Forward: ATCCAACAGCATTTCCAACT<br>Reverse: CAATTAAAACGGAATGTCCA<br>Nested forward: ACCACGTGACGAGGGATAAG<br>Nested reverse: AAAACGGAATGTCCAAATCG	1015	V552I	(Mint Deida et al., 2018)

References

Chung, D. Il, Jeong, S., Dinzouna-Boutamba, S.D., Yang, H.W., Yeo, S.G., Hong, Y. et al. (2015). Evaluation of single nucleotide polymorphisms of pvmdr1 and microsatellite genotype in *Plasmodium vivax* isolates from Republic of Korea military personnel. *Malar. J.* 14, 336. doi:10.1186/s12936-015-0845-6.

Huang, B., Deng, C., Yang, T., Xue, L., Wang, Q., Huang, S. et al. (2015). Polymorphisms of the artemisinin resistant marker (K13) in *Plasmodium falciparum* parasite populations of Grande Comore Island 10 years after artemisinin combination therapy. *Parasites and Vectors*. 8, 634. doi:10.1186/s13071-015-1253-z.

Mint Deida, J., Ould Khalef, Y., Mint Semane, E., Ould Ahmedou Salem, M.S., Bogreau, H., Basco, L. et al. (2018). Assessment of drug resistance associated genetic diversity in Mauritanian isolates of *Plasmodium vivax* reveals limited polymorphism. *Malar. J.* 17(1), 416. doi:10.1186/s12936-018-2548-2.

Naoshima-Ishibashi, Y., Iwagami, M., Kawazu, S. ichiro, Looareesuwan, S. and Kano, S. (2007). Analyses of cytochrome b mutations in *Plasmodium falciparum* isolates in Thai-Myanmar border. *Travel Med. Infect. Dis.* 5(2), 132–134. doi:10.1016/j.tmaid.2006.07.002.

Pearce, R.J., Drakeley, C., Chandramohan, D., Mosha, F. and Roper, C. (2003). Molecular determination of point mutation haplotypes in the dihydrofolate reductase and dihydropteroate

synthase of *Plasmodium falciparum* in three districts of Northern Tanzania. *Antimicrob. Agents Chemother.* 47(4), 1347–54. doi:10.1128/AAC.47.4.1347-1354.2003.

Schwöbel, B., Alifrangis, M., Salanti, A. and Jelinek, T. (2003). Different mutation patterns of atovaquone resistance to *Plasmodium falciparum* in vitro and in vivo: Rapid detection of codon 268 polymorphisms in the cytochrome b as potential in vivo resistance marker. *Malar. J.* 2, 5. doi:10.1186/1475-2875-2-1.

Yan, H., Kong, X., Zhang, T., Xiao, H., Feng, X., Tu, H. et al. (2020). Prevalence of *Plasmodium falciparum* Kelch 13 (PfK13) and Ubiquitin-Specific Protease 1 (pfubp1) Gene Polymorphisms in Returning Travelers from Africa Reported in Eastern China. *Antimicrob. Agents Chemother.* 64(11), e00981-20. doi:10.1128/AAC.00981-20.

* Reference [66] is cited in the Supplementary Materials.
