# Supplementary material for: Molecular Profiles of Multiple Antimalarial Drug Resistance Markers in Plasmodium falciparum and Plasmodium vivax in the Mandalay Region, Myanmar
Source: Microorganisms. 2022 Oct 13;10(10):2021. doi: 10.3390/microorganisms10102021 (PMC9612053; doi:10.3390/microorganisms10102021)
Supplement: Supplementary file 1 [file microorganisms-10-02021-s001.zip › Supplement file 2_Table S2.pdf]

Table S2: The distribution of mutant haplotypes of antimalarial drug resistance between each township in Myanmar *P. falciparum* and *P. vivax* isolates

	Mutant haplotypes	Naung Cho (*n* = 47)	Pyin Oo Lwin (*n* = 6)	Mandalay (*n* = 18)	Tha Beik Kyin (*n* = 24)
pfdhfr	IRNL	23	5	8	9
	IRNI	15	0	6	0
	NRNL	6	1	3	13
	NRNI	2	0	1	2
	NCSI(WT)	1	0	0	0
Chi-square (CI 95%)	25.76				
P value	0.0016				
Degree of freedom (df)	12				

	Mutant haplotypes	Naung Cho (*n* = 46)	Pyin Oo Lwin (*n* = 6)	Mandalay (*n* = 18)	Tha Beik Kyin (*n* = 23)
pfdhps	AGEGA	3	0	0	0
	AGEAA	22	3	6	17
	SGEGA	17	3	4	5
	SGNGA	0	0	1	0
	AGNAA	0	0	1	1
	SGEAA	1	0	3	0
	SGKGA	1	0	2	0
	SGKAA	0	0	1	0
	SAKAA(WT)	2	0	0	0
Chi-square (CI 95%)	34.55				
P value	0.0754				
Degree of freedom (df)	24				

	Mutant haplotypes	Naung Cho (n = 54)	Pyin Oo Lwin (n = 6)	Mandalay (n = 20)	Tha Beik Kyin (n = 16)
pfmdr1	NEFIFD	4	0	0	0
	YEYSFD	1	0	0	0
	NKYSFD	1	0	2	0
	NEFSFD	11	0	5	7
	NEYSYD	17	0	4	2
	NEYSFD(WT)	20	6	9	7
Chi-square (CI 95%)	20.86				
P value	0.1414				
Degree of freedom (df)	15				

	Mutant haplotypes	Naung Cho (n = 43)	Pyin Oo Lwin (n = 5)	Mandalay (n = 18)	Tha Beik Kyin (n = 23)
pfcrt	CIET	43	5	18	22
	CTET	0	0	0	1
Chi-square (CI 95%)	2.902				
P value	0.407				
Degree of freedom (df)	3				

	Mutant haplotypes	Naung Cho (n = 40)	Pyin Oo Lwin (n = 5)	Mandalay (n = 9)	Tha Beik Kyin (n = 21)
pfk13	INYRIRPC	9	0	3	2
	FHYRIRPC	1	0	0	0
	FNYRIHPC	0	0	1	0
	FNYRIRLC	4	0	1	0
	FNYRIRPC(WT)	26	5	4	19
Chi-square (CI 95%)	16.86				

P value	0.1549
Degree of freedom (df)	12

	Mutant haplotypes	Naung Cho (n = 71)	Mandalay (n = 10)	Tha Beik Kyin (n = 12)
pvdhfr	IRMT	24	6	1
	LRMT	16	3	6
	FRTN	8	0	3
	FRTT	0	1	1
	FSMN	1	0	0
	FSTN	12	0	0
	FSTT	2	0	0
	FSTS(WT)	8	0	1
Chi-square (CI 95%)	22.62			
P value	0.0668			
Degree of freedom (df)	14			

	Mutant haplotypes	Naung Cho (n = 57)	Mandalay (n = 9)	Tha Beik Kyin (n = 12)
pvdhps	AGKGV	2	0	1
	SGKGV	41	6	7
	AGKAV	1	3	0
	SGKAV	5	0	4
	SAKAV(WT)	8	0	0
Chi-square (CI 95%)	26.41			
P value	0.0009			
Degree of freedom (df)	8			

	Mutant haplotypes	Naung Cho (n = 68)	Mandalay (n = 10)	Tha Beik Kyin (n = 12)
pvmdr1	FL	9	0	0
	YL	25	2	3
	YF(WT)	34	8	9
Chi-square (CI 95%)	6.141			
P value	0.1889			
Degree of freedom (df)	4			
