# Supplementary material for: Molecular Profiles of Multiple Antimalarial Drug Resistance Markers in Plasmodium falciparum and Plasmodium vivax in the Mandalay Region, Myanmar
Source: Microorganisms. 2022 Oct 13;10(10):2021. doi: 10.3390/microorganisms10102021 (PMC9612053; doi:10.3390/microorganisms10102021)
Supplement: Supplementary file 1 [file microorganisms-10-02021-s001.zip › Supplement file 3_Table S3.pdf]

Table S3: Minor mutations identified in drug resistance genes of Myanmar *P. falciparum*

pfdhfr (*n* = 95)

Mutations	No. of isolates	Frequency (%)	Mutations	No. of isolates	Frequency (%)
Y12C	1	1.1	R129K	1	1.1
I14V	1	1.1	R129G	1	1.1
C18R	1	1.1	K132R	1	1.1
K23R	1	1.1	F136L	1	1.1
E30G	1	1.1	V140A	1	1.1
K49E	1	1.1	E147G	1	1.1
T62K	1	1.1	G154E	1	1.1
V65A	1	1.1	C161R	1	1.1
Y75H	1	1.1	C161W	1	1.1
R77G	1	1.1	G165E	1	1.1
K79R	1	1.1	G165A	1	1.1
S95P	1	1.1	V169A	1	1.1
K97R	1	1.1	Y170H	2	2.1
N100D	1	1.1	I179V	2	2.1
K114Q	1	1.1	I187M	1	1.1
S120C	1	1.1	I187V	1	1.1
V125I	1	1.1			

pfdhps (n = 93)

Mutation	No. of isolates	Frequency (%)	Mutation	No. of isolates	Frequency (%)
I431V	1	1.1	V527G	1	1.1
I441L	1	1.1	H536R	1	1.1
I446V	1	1.1	N546T	1	1.1
W463R	1	1.1	E556G	1	1.1
N464D	1	1.1	R571G	1	1.1
I466M	1	1.1	I572M	1	1.1
C473R	1	1.1	A581E	1	1.1
I479V	1	1.1	K582R	1	1.1
K490E	1	1.1	K583R	1	1.1
C492R	1	1.1	R610G	1	1.1
D499V	1	1.1	M616T	1	1.1
C507R	1	1.1	N620H	1	1.1
E512G	1	1.1	N635D	1	1.1

pfmdr-1 (n = 96)

Mutation	No. of isolates	Frequency (%)	Mutation	No. of isolates	Frequency (%)
I35T	1	1.0	M993V	1	1.0
K36E	1	1.0	C1006R	1	1.0
F42L	1	1.0	R1022G	4	4.2
L44F	9	9.4	S1034I	4	4.2
P45R	9	9.4	D1062V	1	1.0
I59V	1	1.0	K1065E	1	1.0
T70I	1	1.0	S1087P	4	4.2
N84S	1	1.0	A1090T	1	1.0
M85T	1	1.0	I1115T	1	1.0
N86S	1	1.0	K1129N	7	7.3
L87S	1	1.0	P1142S	1	1.0
G88S	1	1.0	Y1178C	1	1.0
V118L	1	1.0	I1186K	2	2.1
L124S	1	1.0	T1192A	3	3.1
E130V	1	1.0	D1196N	1	1.0
L151S	2	2.1	Q1198R	1	1.0
S153P	5	5.2	N1203Y	1	1.0
Y158H	1	1.0	F1213L	1	1.0
L181S	1	1.0	N1229S	6	6.3
P203R	1	1.0	D1236G	1	1.0
M993T	2	2.1			

pfcrt (*n* = 89)

Mutation	No. of isolates	Frequency (%)	Mutation	No. of isolates	Frequency (%)
I59T	2	2.2	I77M	1	1.1
I59V	4	4.5	A79V	1	1.1
S70R	2	2.2	R81K	2	2.2
V71A	1	1.1	T82I	1	1.1
V73A	1	1.1	S90C	1	1.1

pfk13 (*n* = 75)

Mutation	No. of isolates	Frequency (%)	Mutation	No. of isolates	Frequency (%)
V445I	1	1.3	L571P	1	1.3
C447R	1	1.3	S577P	1	1.3
F483S	1	1.3	D584N	1	1.3
F491S	1	1.3	N594S	1	1.3
V494A	1	1.3	E596A	1	1.3
F495L	1	1.3	V603I	1	1.3
E509G	1	1.3	L631H	1	1.3
L514F	1	1.3	V650I	1	1.3
S521P	1	1.3	E651A	1	1.3
C532R	1	1.3	G665S	1	1.3
T535A	2	2.5	A676D	2	2.5
N537S	1	1.3	L678S	1	1.3
I540V	1	1.3			
K563N	1	1.3			

Pfubp-1 (n = 58)

Mutation	No. of isolates	Frequency (%)	Mutation	No. of isolates	Frequency (%)
H1459P	1	1.7	E1530V	1	1.7
H1459R	2	3.4	N1542D	1	1.7
W1470C	2	3.4	H1544R	5	8.6
Y1473H	2	3.4	H1544L	1	1.7
E1509del	3	5.2	H1544Q	1	1.7
D1522G	1	1.7	D1546V	1	1.7
E1530D	25	43.1			

pfcytb (n = 102)

Mutation	No. of isolates	Frequency (%)	Mutation	No. of isolates	Frequency (%)
F30S	1	1.0	T165A	1	1.0
I41T	1	1.0	V171A	1	1.0
G43S	1	1.0	I183N	1	1.0
Y59C	1	1.0	184A	1	1.0
H63Y	1	1.0	185S	1	1.0
E67D	1	1.0	F186S	1	1.0
G71E	1	1.0	F186M	1	1.0
G71R	1	1.0	L191S	1	1.0
Y76C	1	1.0	G195D	1	1.0
F86S	1	1.0	T204I	1	1.0
F86L	1	1.0	I227T	1	1.0
G96E	1	1.0	F235L	1	1.0
Y103C	1	1.0	V274I	2	2.0
L106S	1	1.0			
S107L	1	1.0			
I113V	1	1.0			
F115I	1	1.0			
