# Supplementary material for: Molecular Profiles of Multiple Antimalarial Drug Resistance Markers in Plasmodium falciparum and Plasmodium vivax in the Mandalay Region, Myanmar
Source: Microorganisms. 2022 Oct 13;10(10):2021. doi: 10.3390/microorganisms10102021 (PMC9612053; doi:10.3390/microorganisms10102021)
Supplement: Supplementary file 1 [file microorganisms-10-02021-s001.zip › Supplement file 4_Table S4.pdf]

Table S4: Minor mutations identified in drug resistance genes of Myanmar *P. vivax*

pvdhfr (*n* = 93)

Mutation	No. of isolates	Frequency (%)	Mutation	No. of isolate	Frequency (%)
C16R	1	1.1	D157G	1	1.1
T35A	1	1.1	Y168H	1	1.1
N50S	1	1.1	K169R	1	1.1
V52I	1	1.1	G174R	1	1.1
E66G	1	1.1	G175R	1	1.1
E83G	1	1.1	N186D	1	1.1
D90G	1	1.1	F193Y	1	1.1
N91S	1	1.1	I196T	1	1.1
D105N	1	1.1	A199V	1	1.1
D105G	1	1.1	Y200C	1	1.1
L107M	1	1.1	F206L	1	1.1
G114E	1	1.1	F206Y	1	1.1
W118R	1	1.1	D210G	1	1.1
R131G	1	1.1	E211K	1	1.1
N133D	1	1.1	S218P	1	1.1
K142R	1	1.1	K226R	1	1.1
K148E	1	1.1			

pvdhps (*n* = 78)

Mutation	No. of isolates	Frequency (%)	Mutation	No. of isolates	Frequency (%)
V386M	1	1.3	Q558R	2	2.6
C414R	1	1.3	K561T	1	1.3
C422Y	1	1.3	P573L	2	2.6
R430K	1	1.3	F575L	3	3.8
N432S	1	1.3	F582I	1	1.3
Q434R	1	1.3	R599G	1	1.3
K448N	1	1.3	G612D	1	1.3
I453V	1	1.3	A647V	8	10.3
I453T	1	1.3	Q656R	1	1.3
D459N	1	1.3	R659G	1	1.3
G467D	1	1.3	S663N	1	1.3
H481R	1	1.3	S663G	1	1.3
Y496H	1	1.3	M665L	2	2.6
R504G	1	1.3	R666C	1	1.3
D517G	1	1.3	D668G	1	1.3
S521R	1	1.3	K669E	1	1.3
N537S	1	1.3	L672F	1	1.3
K555R	1	1.3			

pvmdr-1 (n = 90)

Mutation	No. of isolates	Frequency (%)	Mutation	No. of isolates	Frequency (%)
L935S	1	1.1	Y1009H	1	1.1
N943S	1	1.1	I1015T	1	1.1
F947L	1	1.1	E1025V	1	1.1
L953P	1	1.1	N1029S	1	1.1
F954L	2	2.2	I1035V	1	1.1
F962S	1	1.1	D1040G	1	1.1
F964I	1	1.1	N1079S	1	1.1
V968A	1	1.1	F1088I	1	1.1
F983L	1	1.1	I1090N	1	1.1
R986G	2	2.2	I1090T	1	1.1
D994G	3	3.3	R1092K	1	1.1
K997R	1	1.1	V1096G	2	2.2
N1001D	1	1.1	L1104S	15	16.7
A1006T	1	16.7	F1107L	15	16.7

pvk12 (n = 61)

Mutation	No. of isolates	Frequency (%)	Mutation	No. of isolates	Frequency (%)
I380T	1	1.6	C566Y	1	1.6
F381L	1	1.6	A568V	1	1.6
L400P	1	1.6	F569S	1	1.6
E417V	1	1.6	Y574C	1	1.6
E417K	1	1.6	L584P	1	1.6
G422S	1	1.6	L584Q	1	1.6
G435S	1	1.6	I587V	1	1.6
E441G	1	1.6	E588Q	1	1.6
M461I	1	1.6	M594V	1	1.6
K466E	1	1.6	N595S	1	1.6
G470R	1	1.6	F600Y	1	1.6
S471N	1	1.6	S635P	1	1.6
V473A	1	1.6	Q638R	1	1.6
F481S	1	1.6	Y639H	1	1.6
G483E	1	1.6	F642Y	1	1.6
D502G	1	1.6	L649I	1	1.6
N511I	1	1.6	N650K	1	1.6
N517S	1	1.6	K655Q	1	1.6
G530S	1	1.6	K656E	1	1.6
S536P	1	1.6	M657T	1	1.6
E542K	1	1.6	F659I	1	1.6
A555T	1	1.6	A661V	1	1.6
P556L	1	1.6	A662T	1	1.6
S562P	1	1.6	I670T	1	1.6
S562F	1	1.6	N675K	1	1.6
C566R	1	1.6	D688N	10	16.4
